# Care provided by general practitioners to patients with psychotic disorders: a cohort study

**DOI:** 10.1186/1471-2296-11-92

**Published:** 2010-11-25

**Authors:** Marian JT Oud, Jan Schuling, Klaas H Groenier, Peter FM Verhaak, Cees J Slooff, Janny H Dekker, Betty Meyboom-de Jong

**Affiliations:** 1 Department of General Practice, University Medical Centre Groningen, University of Groningen, Antonius Deusinglaan 1, 9713 AV Groningen, The Netherlands; 2 NIVEL, the Netherlands Institute for Health Services Research, Utrecht, The Netherlands; 3 Department of Psychiatry, University Medical Centre Groningen, Groningen, The Netherlands; 4 Mental Health Centre Drenthe, Assen, The Netherlands

## Abstract

**Background:**

Patients suffering from psychotic disorders have an increased risk of comorbid somatic diseases such as cardiovascular disorders and diabetes mellitus. Doctor-related factors, such as unfamiliarity with these patients, as well as patient-related factors, such as cognitive disturbance and negative symptoms, contribute to suboptimal health care for these patients.

General practitioners (GPs) could play a key role in diagnosing and treating this somatic comorbidity as in the Netherlands, almost all residents are registered at a general practice. This study aims to find out whether there are any differences between the levels of health care provided by GPs to patients with psychotic disorders, compared to other types of patients.

**Methods:**

A cohort of patients with an ICPC code of psychosis and two matched control groups, one consisting of patients with other mental problems and the other one of patients without any mental problems, were followed over a period of 5 years.

**Results:**

Patients with psychotic disorders (N = 734) contacted the GP practice more often than patients in the control groups. These patients, both adults (p = 0.051) and the elderly (p < 0.005), received more home visits from their GPs. In the adult group (16 to 65 years old inclusive), the number of consultations was significantly higher among both psychosis patients and the group of patients with other mental problems (p < 0.0005). The number of telephone consultations was significantly higher in both age categories, adult group (p < 0.0005), and > 65 years old (p = 0.007). With regard to chronic illnesses, elderly psychosis patients had fewer contacts related to cardiovascular diseases or chronic lung diseases.

**Conclusion:**

Patients with psychotic disorders contact the GP practice more frequently than other types of patients. Adult psychosis patients with diabetes mellitus, cardiovascular diseases or chronic lung diseases receive the same amount of health care for these diseases as other primary care patients. The finding that older patients with psychotic disorders are diagnosed with cardiovascular diseases and obstructive lung diseases less frequently than other types of elderly patients requires further study.

## Background

Psychotic disorders are severe psychic disorders involving a loss of contact with reality. Medical signs can vary greatly, including both acute and chronic phases. Patients suffering from psychotic disorders are faced with a large spectrum of comorbid diseases [1,2]. This has been demonstrated most clearly for obesity and related conditions such as diabetes mellitus and the metabolic syndrome [3-6], and for cardiovascular diseases [7,8]. There are also indications that psychosis patients are more susceptible to lung diseases, thyroid diseases, and infectious diseases such as tuberculosis and hepatitis B and C [9,10]. This somatic comorbidity can be explained by several, partly related, causes. Schizophrenic patients more often have an unhealthy lifestyle: they move less, eat poorly, smoke more often, use cannabis and other narcotic drugs, and drink alcohol more often [11,12]. In addition, antipsychotic drug treatment also contributes to an increase in weight and the development of the metabolic syndrome [13,14].

Consulting a doctor can sometimes constitute a barrier to psychosis patients: these patients might observe and interpret physical complaints differently and deal with pain differently [15,16]. Moreover, some patients find it hard to discuss their problems with others [17]. In many cases, including acute illnesses, psychosis patients tend to seek help at a later stage or, indeed, when it is too late [18,19].

In the Netherlands, under the Care Insurance Act, the more than 16 million residents - excluding military personnel and inhabitants of nursing homes and mental hospitals - are registered with a general practitioner practice. From the residents with severe mental disorders, there are about 18.000 patients hospitalised where they receive the somatic care from a somatic physician in the hospital.

The duty of general practitioners (GPs) is to act adequately and professionally in case of questions from patients regarding their health problems. There is an assumption that they are well informed about somatic and psychiatric morbidity and comorbidity, and that they document such information in the electronic medical record. They carry out diagnostics and treatment and guide chronically ill people. One of their tasks is detecting and treating health problems at an early stage. According to the literature, however, GPs pay less attention to detecting risk factors for cardiovascular diseases, and undertreat hypertension, diabetes mellitus and fat metabolism disorders in cases of patients with psychotic disorders [20,21]. This raises questions regarding the quality of care provided by Dutch general practitioners to patients with psychotic disorders. To this purpose, the following research questions have been formulated:

- Is there any difference between the use of care by patients with psychotic disorders and other patients at a GP practice?

- Is there any evidence that general medical care provided to patients with psychotic disorders in the case of chronic illnesses, such as diabetes mellitus, cardiovascular diseases, and obstructive lung diseases, differs from the type of care offered to other patients?

## Methods

Longitudinal registration data, over a period of five years, taken from the electronic patient records of the Netherlands Information Network of General Practice (LINH) were used. LINH covers 89 automated general practitioner practices with nearly 340.000 registered patients who are representative of the Dutch population concerning age, sex, and urbanisation degree. LINH collects data about morbidity, contacts, drug prescriptions, and referrals. The selection of patients from this database was based on codes of the International Classification of Primary Care (ICPC) assigned by general practitioners. The registration period for research purposes was 1 January 2002 to 1 January 2007.

Three groups of patients were selected based on ICPC codes. The index group of patients with psychotic disorders (N = 734) consisted of patients who had contact with the GP practice between 1 January 2002 and 31 December 2002 for the following ICPC codes: P71 Organic psychoses, P72 All types of schizophrenia, P73 Affective psychoses, and P98 Non-specific psychoses. The control groups had the same sizes (N = 734) and had been matched according to age and gender. One control group, the 'Control with P' group, consisted of patients who had consulted their general practitioner about other (all remaining) mental health problems and diagnoses (P01-P29, P70, and P74-P97) in 2002. Meanwhile, the other control group, the 'Control without P' group, consisted of patients who had visited their general practitioner without any mental health problems in 2002.

The International Classification of Primary Care (ICPC) classifies a delirium as an organic psychosis. As this specific condition occurs mostly in elderly patients, we chose for an analysis of our data in two age categories. The group of adults (aged 16 to 65 inclusive) consisted of 532 patients (72.5%) and the group of elderly (aged > 65) consisted of 202 patients (27.5%) of the total group (N = 734). These categories will be referred to as 'adults' and 'elderly' respectively for the remainder of the paper.

The morbidity database contains all contacts with the general practitioner practice, irrespective of the nature of these contacts, and each contact is linked to an episode with an ICPC code. An episode is a period of care containing all contacts concerning a specific symptom or illness [22], which can be continuous in chronic diseases like diabetes, or of limited time period as in an acute disease, like tonsillitis.

Care provided by the general practitioner in case of diabetes mellitus, cardiovascular diseases, and chronic respiratory diseases was measured by means of the number of contacts and the referrals. Contact frequencies and incidences were converted into numbers per 1000 patient years. In order to assess any difference in outcome between the three groups, the Kruskal-Wallis test was used. The differences between every two groups were compared using the Mann-Whitney test.

The study was carried out according to Dutch legislation on privacy. The Dutch Data Protection Authority approved the privacy regulation of the study. According to the Central Committee on Research Involving Human Subjects (CCMO, http://www.ccmo-online.nl/main.asp?pid=1&taal=1 webcite), obtaining informed consent is not obligatory for observational studies.

## Results

The group of patients with psychotic disorders (N = 734) consisted of 315 men and 419 women. A total of 532 of them were aged 16-65 and 202 of them were over 65 years of age. The average age of males was 48 and the average age of females was 54. The nature of the psychotic disorders is described in table 1.

**Table 1:** Patients with psychotic disorders (N = 734)

	*aged 16-65*	*aged > 65*	*total*
Organic psychosis (P71)	67 (12.6%)	111 (53.6%)	178

Schizophrenia (P72)	148 (27.8%)	26 (12.6%)	174

Affective psychosis (P73)	196 (36.8%)	32 (15.5%)	228

Non-specific psychosis (P98)	175 (32.9%)	38 (18.4%)	213

**Total**	**532 (100%)**	**207 (100%)**	739*

* 5 patients with two diagnoses in 2002

On average, patients with psychotic disorders stayed in a GP practice for 4,0 years. This was 4,4 years for patients in the 'Control with P' group, and 4,6 years for patients in the 'Control without P' group. During the research period, various ICPC codes of psychotic disorders were registered with 56 patients in the P group: 53 patients were diagnosed with two disorders and three patients with three.

### Contacts with the general practitioner practice

#### Total number of contacts

Patients with psychotic disorders contacted the GP practice more often compared to patients in the control groups (p < 0.0005). The entire morbidity database consisted of 52.699 contacts. The group of 'Psychotic disorders' made up 22.691 of these contacts, of which 5.553 contacts resulted from an episode of psychosis. The 'Control with P' group had 20.420 contacts, and the 'Control without P' group 9.588.

Converted into 1000 patient years, the group of 'Psychotic disorders' had an average of 2,63 contacts per episode, which equals 2.093 episodes. The 'Control with P' group had an average of 2,17 contacts per episode, totalling 2.314 episodes. Finally, the 'Control without P' group had an average of 1,84 contacts per episode, representing 1.354 episodes.

#### Types of contacts

Patients with psychotic disorders, both adults (p = 0.051) and elderly (p < 0.005), received relatively more home visits from their general practitioner. See table 2. Additionally, out of hours home visits were made more often to adults with psychotic disorders than to controls, though these differences were not statistically significant. The number of consultations at the office, made by adults with mental disorders ('P group and 'Control with P' group), was significantly higher than 'Control without P' (p < 0.0005), but this was not the case for elderly patients. The number of telephone consultations was significantly higher in both age categories, of both groups of patients with mental disorders: adults (p < 0.0005) and elderly (p = 0.007).

**Table 2:** Nature of contacts (absolute numbers per 1000 patient years)

Daytime	*age*	*Psychosis*	*Control P*	*Control w P*	*p value*
visit	16-65	151	62	23	0.051
	> 65	323	283	157	**<0.0005**

consultation	16-65	1915	1716	951	**<0.0005**
	> 65	437	601	583	0.272

phone	16-65	556	491	227	**<0.0005**
	> 65	280	292	184	**0.007**

**ENW•**					

visit	16-65	12	6	2	0.876
	> 65	16	20	5	0.544

consultation	16-65	31	37	12	0.432
	> 65	6	4	6	0.447

phone	16-65	74	21	7	0.122
	> 65	13	16	2	0.779

• ENW = evening, night, weekend

### Chronic illnesses

The three groups of adults showed no differences with respect to the number of episodes with chronic illnesses (table 3). Elderly with psychotic disorders had fewer episodes with cardiovascular diseases than the peer groups of 'control with P' and 'control without P' (p < 0.0005); this also applies to the number of episodes with asthma/COPD (p < 0.007) (table 3). The number of episodes with diabetes mellitus did not vary among the three groups of older patients.

**Table 3:** Chronic illnesses per 1000 patient years

Chronic illness	*age*	*Psychosis*	*Control P*	*Control w P*	*p value*
Diabetes mellitus	16-65	12.61	9.57	5.94	0.320

Diabetes mellitus	> 65	9.20	10.28	7.39	0.220

Cardiovascular diseases	16-65	24.29	29.98	18.62	0.498

Cardiovascular diseases	> 65	24.14	48.28	36.61	**<0.0005**

Asthma/COPD	16-65	11.12	15.11	6.60	0.414

Asthma/COPD	> 65	3.19	10.97	4.07	**0.007**

### Contacts and referrals per chronic illness

#### Diabetes mellitus

The average number of contacts per patient during the registration period was highest for psychosis patients, but the differences between the three groups were not significant (table 4). The number of referrals to an internist was low, but patients with mental disorders were referred more often. Patients with psychotic disorders were sent more frequently to an eye doctor or a podiatrist (table 5). Due to small numbers, there are no statistically significant differences between the various groups, however.

**Table 4:** Contacts per chronic illness during the registration period per 1000 patient years

		*Contacts per 1000* *patient years*	*p value*
Aged 16 to 65 inclusive			
Diabetes mellitus	Psychosis	107	
	Control P	101	0.300
	Control w P	33	

Cardiovascular diseases	Psychosis	175	
	Control P	194	0.540
	Control w P	133	

Asthma/COPD	Psychosis	41	
	Control P	64	0.388
	Control w P	21	

**Aged > 65**			

Diabetes mellitus	Psychosis	88	
	Control P	130	0.232
	Control w P	64	

Cardiovascular diseases	Psychosis	134	
	Control P	272	**<0.0005**
	Control w P	186	

Asthma/COPD	Psychosis	17	
	Control P	64	**0.008**
	Control w P	27	

**Table 5:** Referrals per chronic illness in absolute numbers

	*Psychosis*	*Control P*	*Control w P*	*Total*
**Diabetes Mellitus**				
Internist	6	5	2	13
Eye doctor	28	11	8	47
Dietician	3	0	5	8
Podiatrist	41	32	22	95

**Cardiovascular diseases**				
Cardiologist	18	12	7	37

**Asthma/COPD**				
Lung specialist	3	11	4	18

#### Cardiovascular diseases

Elderly people with psychotic disorders contacted their GP related to cardiovascular diseases less frequently. In contrast, elderly 'control with P' patients had more contacts than the others (table 4). Psychosis patients were referred to a cardiologist more often (table 5). Because of small numbers, again there are no statistically significant differences between the various groups.

#### Asthma/COPD

Elderly with psychotic disorders were checked for chronic lung diseases less frequently, and were referred less frequently to a pulmonary specialist. However, as with cardiovascular diseases, elderly 'control with P' patients had contact with their general practitioner more frequently (table 5). Due to low numbers, there was no statistically significant difference, however.

## Discussion

This research shows that patients with psychotic disorders frequently contact their GPs. In particular, the age group 16 - 65 years receives a lot of care in comparison to the control groups: there are more consultations at the office, GPs pay them more visits, and have more telephone contacts. As people become older, the percentage of organic psychoses increases to more than fifty percent. Mental confusion and hallucinations in the elderly, whose brains are more vulnerable, are often caused by physical disorders [23]. As a consequence, the elderly have a different spectrum of psychotic disorders than younger adults or adolescents. Organic psychosis dominates in this age group, and accordingly these patients receive more home visits from their GPs.

A minority (7.6%) of the patients of the group with psychotic disorders have been registered with different diagnostic labels during the five registration years. A diagnosis of 'non-specific psychosis' (code P98) can typically change into 'schizophrenia' (P72) or 'affective psychosis' (P73). On occasion, the difference between these diagnoses is unclear.

Chronic diseases, notably diabetes mellitus, cardiovascular diseases and chronic lung diseases, are frequent in patients with psychotic disorders, although in this research the incidence of these chronic diseases in adults with psychotic disorders does not differ significantly from the other groups. This deviating finding could be an effect of the sample group, which contained a wide variety of psychotic disorders. There might also be cases of underdiagnosis, which is in keeping with the results of other research [20], because the recognition of the high risk of these chronic diseases in patients with psychotic disorders is rather new for GPs. In addition to underdiagnosis, a further problem in relation to these conditions is undertreatment [20,21,24]. However, in this Dutch study the number of contacts made by the GP, related to diabetes mellitus, is similar for the psychosis group and the control groups.

A small number of patients with diabetes mellitus is referred to an internist. This number is almost equal for both groups with mental health problems. On the other hand, psychosis patients are referred more often to an eye doctor or a podiatrist. An explanation could be that the treatment targets were not met by the GP as a consequence of non-compliance in these patients. The high number of referrals to the eye doctor could also be biased by the occurrence of other eye diseases, because patients with schizophrenia attend visual examinations less frequently than others, and their vision is notably weaker [25]. Finally, these patients might be more severely ill because they contact their GP at a later stage of the disease [17-19].

With respect to cardiovascular diseases, the average number of GP contacts per episode for patients aged 16 to 65 inclusive also appears to be the same as for the control groups, although elderly psychosis patients are seen far less frequently for cardiovascular checks. We presume that the GP's care for these elderly patients focuses more on the quality of life than on diagnosing and treating risk factors for cardiovascular diseases. Also, the cardiovascular patients with psychotic disorders are referred to secondary care more often. These patients suffer from more additional risk factors such as obesity, diabetes mellitus and smoking addictions [26,27], which might make them more severely ill.

Among elderly psychosis patients, both the number of episodes and the number of follow-up contacts appears to be lower with respect to chronic lung diseases. A possible explanation for these findings could be that there is some kind of selection due to the early death of schizophrenia patients [28,29].

GPs aim to meet the demands of the patient. However, providing care to patients with psychotic disorders is complicated. These patients find it difficult to carry out the doctor's lifestyle advice and their adherence to prescribed treatment is low. There are often severe psychosocial problems, requiring their attention and inhibiting life style changes. On the other hand, these patients do use the primary care very frequently, and are often satisfied with the amount and type of service provided for their physical needs [30]. Considering the high contact frequency, the GP gets ample opportunities to perform regular health checks. One can assume that active screening for diabetes mellitus, cardiovascular diseases, and obstructive lung diseases in these patients will lead to more diagnoses. Starting treatment and coaching at an earlier stage could delay the progress of the illnesses [31].

Since the study period (2002-2007), primary care for chronic diseases, such as diabetes mellitus, cardiovascular diseases, and chronic pulmonary diseases, has developed into programmatic care, through which patients are contacted by a practice nurse and undergo a physical and laboratory check. A great deal of time and attention is spent on education, counselling, and improving self-management. Patients with psychotic disorders also deserve this type of care.

It is clear that the division between mental health care and primary care hinders the provision of effective, coherent care. Therefore, the European Psychiatric Association (EPA) and the World Psychiatric Association (WPA) have stated that collaboration and communication between GPs and psychiatric services should be promoted [32,33]. Coordination between all care providers within the family practice and with specialist services is essential for optimal patient care.

### Strong and weak points

The LINH database has supplied longitudinal data about morbidity and the use of care in Dutch general practitioner practices. The index group of 'Psychotic disorders' covers the entire range of psychotic disorders in the general practitioner coding system (ICPC).

Comparing patients suffering from psychotic disorders with both patients suffering from other mental health disorders and patients without mental health problems has produced a clear picture of the type of care provided to psychotic patients. This research only involved those patients whose psychotic disorder had been registered in the electronic medical record by the general practitioner and who had visited their general practitioner in 2002. This means that all patients whose psychosis had not been recognized and registered, as well as those who had not visited their general practitioner in 2002, were excluded from this research.

## Conclusions

Patients with psychotic disorders contact their general practitioner practice more often than other types of patients. Adult psychosis patients with diabetes mellitus, cardiovascular diseases or chronic lung diseases receive the same amount of care from the GP as other patients. Elderly patients have a different spectrum of psychotic disorders, with less frequent diagnosis of cardiovascular diseases and obstructive lung diseases in comparison to other elderly patients. Moreover, these diseases, once diagnosed, are checked less frequently among this group.

### Recommendations

Patients with psychotic disorders could gain a health benefit if GPs performed annual health check-ups, paying attention to risk factors for cardiovascular diseases, eating patterns, lung functions, and use of tobacco. The GP should explore barriers to compliance with the patient, while trying to impart the importance of controlling the above risk factors. In order to be able to change unhealthy behaviour patterns, intensive coaching is required. Regular exercise programmes [34], which should be adapted to the individual physical fitness level [35], and meal delivery could be effective. Considering the higher number of referrals, further research should demonstrate whether patients are really faced with a more severe stage of a disease and whether they could benefit from active screening and monitoring.

## Competing interests

The authors declare that they have no competing interests.

## Authors' contributions

MO, JS, and PV designed the study. JS obtained funding for the study. KG performed the statistical analyses. MO drafted the manuscript. All authors read and approved the final manuscript.

## Pre-publication history

The pre-publication history for this paper can be accessed here:

http://www.biomedcentral.com/1471-2296/11/92/prepub
